# Co-occurrence of autoimmune thyroid disease in a multiple sclerosis cohort

**DOI:** 10.1186/1740-2557-2-9

**Published:** 2005-11-09

**Authors:** JS Sloka, Pryse-WEM Phillips, M Stefanelli, C Joyce

**Affiliations:** 1 Department of Neurology, Memorial University of Newfoundland, St John's, Canada; 2 Department of Endocrinology, Memorial University of Newfoundland, St John's, Canada

## Abstract

**Background:**

Multiple sclerosis (MS), Hashimoto's disease and Graves' disease are autoimmune diseases that may share similar pathogenic mechanisms. The co-occurrence rates and demographic characteristics of Graves' disease and Hashimoto's disease (HT) in our MS population are compared with the general population.

**Methods:**

The prevalence of thyroid disease in our MS patients was determined by chart review and survey. Previous diagnosis of thyroid disease, age at diagnosis, treatment used, and about the use of disease modifying medications used to treat their MS were asked. Chart reviews were used to estimate the population prevalence of Graves' disease and Hashimoto's disease and to estimate the demographics of patients with thyroid disease.

**Results:**

A significant co-occurrence of Graves' disease with MS (p = 0.002), and a non-significant co-occurrence of Hashimoto's disease were noted (p = 0.097). No difference in the age of onset or gender of thyroid disease in MS patients compared to the general population was found.

**Conclusion:**

There is a significant co-occurrence in patients with MS and Graves' disease, and a trend to co-occurrence in patients with MS and Hashimoto's disease. There are no differences in the demographics of patients with thyroid disease in our MS patients compared to the general population.

## Introduction

Autoimmune diseases, sometimes defined as *a clinical syndrome caused by the activation of T cells or B cells, or both, in the absence of an ongoing infection or other discernible cause *[1], comprise a heterogeneous group of disorders wherein alterations in the immune system may result in a spectrum of disease that either targets specific organs or affects the body systemically. Even though each purported autoimmune disease affects disparate organs and systems, the existence of fundamental pathophysiological mechanisms is hypothesized. Recent such epidemiological studies have shown an increased statistical susceptibility for people with one autoimmune disease to develop another [2-5]. This provocative increased relative risk of acquiring a second autoimmune disease may be due to a genetic susceptibility that affects both diseases, an environmental trigger that initiates both diseases, the alteration of the body's homeostasis by one disease that creates susceptibility to another, or some as yet undefined shared mechanism.

Some studies have shown that autoimmune diseases "cluster together"[5]. Specifically, several studies have shown an increased co-occurrence of MS with Hashimoto's thyroiditis (HT) as compared to the general population [3,4,6] as well as an increased co-occurrence of MS with Graves' disease [7] while other have not [2]. No comment on the age of onset of thyroid disease in MS patients compared with the general population has been previously made. Some studies have shown that the risk of acquiring autoimmune thyroid disease has been shown to increase after treatment with some MS disease modifying agents [8]. The population co-occurrence of MS and autoimmune thyroid disease has not been studied after accounting for previous disease modifying therapy[5].

We have undertaken an epidemiological study of MS and autoimmune thyroid diseases in Newfoundland and Labrador (NL) to elucidate some of the epidemiological features of any co-occurrence found amongst these diseases. Presumably, if one is susceptible to more than one disease, then features such as clinical onset of disease may be seen earlier in those more susceptible, especially if one disease provokes another.

NL, with its centralized and comprehensive medical records system and its unique population [9,10] is an excellent place to perform such epidemiological studies. The prevalence of MS was previously determined [11] with near complete case ascertainment. However, the prevalence of thyroid disease in the general population of NL was unknown. Therefore, an estimate of the prevalence of thyroid disease in NL was also undertaken in order to compare the prevalence of thyroid disease in the MS population with the prevalence of thyroid disease in the general NL population and to compare their demographics.

## Methods

An incidence and prevalence study for the Canadian province of Newfoundland and Labrador has recently been completed (Dec 31, 2001), demonstrating an MS population prevalence for MS of 94.4 per 100,000 [11]. A database was created with patient information for 493 confirmed living cases [11]. Complete chart reviews were conducted on each confirmed case to determine if these patients had concomitant thyroid disease. Careful chart reviews were performed in the patients' neurology charts and their inpatient hospital charts. As well, electronic searches through the hospital information systems for the four main community hospitals were conducted for both case ascertainment and diagnosis confirmation of MS and thyroid disease. MS diagnosis type, thyroid diagnosis type, and age at onset of disease were captured.

In October 2003, a survey was mailed to the living diagnosed patients from the prevalence study whose current addresses could be confirmed, with a subsequent mailer to those that did not initially respond after 6 weeks. The survey asked questions relating to concomitant thyroid disease and age of initial symptoms (Table 1). This survey and all case searches were conducted with the approval of the Human Investigations Committee of Memorial University.

**Table 1:** Survey questions related to thyroid disease

Have you ever been treated for thyroid disease in the past?		
Yes	No	
If yes (multiple yes answers are possible)		
Was this called Graves' disease?	No	Yes
Was this called Hashimoto's disease?	No	Yes
Was this called hyperthyroidism?	No	Yes
Was this called hypothyroidism?	No	Yes
Did you ever take Synthroid?	No	Yes
Did you ever take Eltroxin?	No	Yes
Did you ever take propylthiouricil?	No	Yes
Did you ever take methimazole?	No	Yes
Did you ever have radiation treatment for your thyroid?	No	Yes
At what age were you diagnosed with any of the above?	________ years old	

To determine the prevalence of thyroid disease in the general population, a representative population sample was sought whereby complete case histories were taken on clinic patients and bias towards a known predisposition to thyroid disease was minimized. The general neurology clinic was used for case ascertainment of thyroid disease in a representative general population after it was determined by thorough literature search that there is no known association of thyroid disease in patients with primary headache disorders or injuries (eg falls, motor vehicle accidents (MVA), sports injuries). Therefore, a retrospective chart review was conducted through all the patient charts that have been evaluated in the general neurology clinic of two of the authors (WPP and MS) over the previous 8 years. Headache type/injury source, gender, age at clinic visit and concomitant thyroid disease were captured.

In order to minimize case ascertainment discrepancies between the MS population sample and the general population sample, a followup electronic chart review was performed on both the general and MS population cases. As per the previous MS incidence and prevalence study, complete physician billing records towards all thyroid disease and MS for the entire province were requested from the provincial medical insurance plan via an official government Order in Council. This aided in the confirmation of thyroid disease in both sample populations and minimized the number of missed thyroid histories in the original chart review. These billing records also increased accuracy of case ascertainment and thus reduced referral and ascertainment bias. Confirmation of thyroid disease in both the MS population and the general population sample was completed for all diagnoses via review of patient charts and electronic records searching specifically for thyroid stimulating hormone (TSH) receptor autoantibody tests, nuclear medicine studies, and diagnosis by an endocrinologist or internist. Patients with MS were excluded from the general population database and patients from St Pierre et Miquelon, a neighbouring territory, were excluded from databases due to inconsistent referral.

The diagnostic criteria used by endocrinologists for Graves' disease has evolved over time[12]. All patients with diagnosed Graves' disease in our study had increased free thyroxine and decreased TSH. All patients were evaluated using either ^99 m^Tc radionucleotide scintigraphy or ^131^I uptake and scan. The diagnosis in some patients was supported by positive TSH receptor autoantibodies. Patients with Graves' disease were usually treated with anti-thyroid drugs, and subsequent radioablation therapy if required. The diagnosis of Hashimoto's disease was based on low or low normal free T_4 _with elevated TSH, and was supported by the presence of elevated anti-thyroperoxidase autoantibodies, and anti-thyroglobulin autoantibodies. In addition, on clinical examination, a firm goiter in the context of the aforementioned laboratory findings corroborated the diagnosis of Hashimoto's disease. These patients were generally treated with thyroid hormone replacement therapy.

Through initial sampling of patient charts, it was estimated that a total of 500 patients would be found. For a estimated prevalence of Hashimoto's thyroiditis of 2% [13] (yielding 10 of 250 patients) and Graves' disease of 0.8% [13,14] (yielding 4 of 250 patients), it was decided that this would not yield a large enough sample size to give a clear indication of the demographics of thyroid disease in NL. Therefore, another retrospective chart review was conducted in order to estimate the demographics of Graves' and Hashimoto's disease. All the endocrinology clinic patient charts of another author (CJ) were reviewed from the previous 15 years to determine the demographics of patients in the province with (autoimmune) thyroid disease not associated with either malignancy or specific genetic syndromes such as Multiple Endocrine Neoplasia II. Patient age at onset, disease type and gender were recorded.

Comparison of prevalences between the MS and General populations were performed both before and after direct age and sex standardization of population samples. This minimized bias due to different population distributions.

Two-tailed t tests were used for comparison of average age of onset of disease. Chi squared tests were used to compare proportions unless samples were less than 5, and Fisher's exact test was used. An α of 0.05 was used for significance.

## Results

A previous MS prevalence study found 493 living patients with MS in NL [11]. In October 2003, 438 addresses were confirmed for a list of living MS patients [11] and these patients were mailed surveys. After one survey mailing and a followup mailing for non-responders, 328 surveys were returned (a 75% rate of return). The results of this survey are shown in Table 2. There were no significant differences in demographics between the patients that returned the surveys and the MS patients in the database.

**Table 2:** Patient characteristics of survey results.

	All MS Patients	Survey Returned	Significance
Average Age	45.7	45.2	p = 0.53
Female to Male Ratio	2.72:1	2.86:1	p = 0.76
Age of First Symptoms	32.2	32.4	p = 0.81
Proportion RRMS	0.83	0.83	p = 0.97

RRMS – Relapsing Remitting Multiple Sclerosis

532 patients with headache and various injuries were found by chart review. The average age was 39.6 years and the female to male ratio is 2.57:1. There were 274 patients with migraines, 25 people with MVAs, 27 with injuries and the rest were of various other headache types. The prevalences of thyroid disease in both the MS population and in our representative sample of the general population are shown in Table 3. There was an increased prevalence of both Graves' disease and Hashimoto's disease in the MS population compared to the general population, although the co-occurrence of Hashimoto's was not significant after age and sex standardization (Table 3).

**Table 3:** Prevalence of thyroid disease in MS patients and in a representative sample of the general population

	MS Population (N = 491)	General Population (N = 532)	Significance after age-sex standardization
Graves'	15 (3.1%)	2 (0.4%)	p = 0.002*
Hashimoto's	26 (5.5%)	12 (2.2%)	p = 0.097

* by Fisher exact test

Access to all provincial records made laboratory confirmation of TSH results through electronic and hospital records thorough. Of the 532 patients used for the representative general population, 472 patients had recently TSH results drawn within the past 9 years, most (433 – 81.4%) within 1 year of their neurology consultation or since. In fact, many have had almost yearly serial TSH tests performed for the past several years. We are unable to speculate why this test appears to be so well-used in this general patient population. Electronic and hospital records were also used to confirm the use of thyroid replacement medications as well as the results of ultrasound and nuclear medicine tests for diagnostic workup of thyroid disease. Records of patient visits to endocrinologists were also available for this confirmation.

From the survey results, the chart reviews and the electronic record searches, 15 patients with Graves' disease and 26 patients with Hashimoto's disease were found in the MS population (Table 4). The age of onset of thyroid disease was confirmed for all MS patients with concomitant dysthyroidism. Neither the age of onset of thyroid disease in the MS and general population nor the male to female ratio were significantly different.

**Table 4:** Demographics of MS patients with thyroid disease and demographics of patients with thyroid disease. Confirmed age means the number of MS patients whose age at onset of thyroid disease could be confirmed.

	MS with Graves'	Graves' Disease	Significance	MS with Hashimoto's	Hashimoto's	Significance
Total Number	15	80		26	57	
Confirmed Age	10			13		
Average Age of Onset of Thyroid Disease	35.2	37.5	p = 0.61	36.2	37.9	p = 0.70
Female:Male	13:2	72:8	p = 0.66*	21:5	48:9	p = 0.63

* by Fisher exact test

The age of onset (age at appearance of first symptoms) of MS was confirmed for all MS patients with concomitant thyroid disease and for all MS patients. The differences between the age of onset of all MS patients and those MS patients with both Graves' (p = 0.81) and Hashimoto's (p = 0.71) was not significant.

Only 1 out of the 10 MS patients with Graves' disease that responded completely on the survey had undertaken treatment with an interferon-β prior to developing dysthyroidism which she had developed at 56 years of age, 1 year after commencing therapy.

## Discussion

Multiple sclerosis (MS) is a chronic inflammatory disease of the central nervous system. It damages both oligodendroglia and axons and may cause paralysis, sensory disturbances, incoordination, visual impairment, and alterations in autonomic and sexual function [15]. Its precise etiology has not been elucidated but many observations suggest that both genetic susceptibility and environmental factors contribute [16]. There continues to be a debate regarding the eligibility of MS as an autoimmune disease [17,18], although it is generally accepted as such [19].

Hashimoto's thyroiditis is an organ-specific autoimmune disease that is the most common cause of goitrous hypothyroidism[20]. Identification as an autoimmune disease follows observations that lymphocytes infiltrate the thyroid [21] and autoantibodies against thyroglobulin, thyroid peroxidase, and (rarely) thyroid hormone stimulating receptor are found in patients with HT[20]. The initiation of the autoimmune events of HT are not precisely known, but could be caused by a molecular mimicry mechanism, abnormal antigen-specific induction of T-cells due to abnormal HLA-related SPC genes, mutation of T cells to form abnormal clones, or an immune defect causing reduced induction of T suppressor cells by specific antigens [22].

Graves' disease is an autoimmune disorder characterized by the production of thyroid-stimulating IgG immunoglobulins directed towards the thyroid simulating hormone (TSH) receptor of the thyroid gland [23]. The exact mechanism for the stimulation of these autoantibodies is unknown, but several have been proposed [24].

There is an increased risk of autoimmune thyroid disease in patients with MS in Newfoundland and Labrador. The study was comparably large (493 MS patients, 532 controls) and there was an excellent survey response rate (75%). There were no gender or age discrepancies in those that responded to the survey (Table 2). Diagnostic confirmation was extensive and case ascertainment was comprehensive. Attempts to reduce reporting and ascertainment bias were made.

Community surveys of hyperthyroidism due to Graves' disease have reported prevalence rates of less than 1.9%, but the prevalence can be as high as 2.7% if subclinical cases are included [13]. Our study showed a rate of 0.38% for Graves' disease (95% CI: 0.07–2.1%) which is compatible with previously reported values. The prevalence of hypothyroidism in other populations may be between 0.2% and 3% [13,25]. Our study showed a rate of 2.3% (95% CI: 1.1–4.9%) in patients with MS, which is again similar to those values reported elsewhere.

Other studies have demonstrated a high prevalence of Graves' disease in first degree relatives of patients with MS [2,7] but not with MS probands (in contrast to our study). Since some MS patients treated with interferon-β may develop laboratory signs of autoimmune thyroid disease [8], it is important to determine whether disease modifying drugs have caused this increased prevalence of Graves' disease in our MS population.

Our survey asked questions relating to previous treatment with disease modifying drugs and their initiation. Only 1 out of the 10 MS patients with Graves' disease that responded completely on the survey had undertaken treatment with an interferon-β prior to developing dysthyroidism which she had developed at 56 years of age, 1 year after commencing therapy. It is unknown whether she would have developed it without the interferon-β, but we can test for significance while excluding her since it is appropriate to look for co-occurrence once other avenues of co-occurrence are factored out[5]. If one only includes the 9 complete respondents via survey who either had not even undertaken interferon-β therapy (N = 5) or had begun therapy after the development of Graves' (N = 4), there is still a significant proportion of MS patients with Graves' disease (p = 0.024).

Although not as significant as the Graves' and MS co-occurrence, the co-occurrence of MS with HT showed a trend towards significance (p = 0.097). This confirms the findings in two previous studies, both of them using laboratory results to show an increase in serum TSH and decrease in T3 and one also using serum autoantibodies [3,6] although other large studies have failed to show an increased risk of other autoimmune diseases (in particular, HT) in multiple sclerosis patients [2,26,27]. The retrospective case ascertainment of HT is most likely subject to greater error than in the case of Graves' disease. Most patients that returned surveys stated that they had primary hypothyroidism, but did not state they had Hashimoto's disease (Table 1). Most charts stated the same. The surveys and charts of subjects with Graves' disease more specifically stated Graves' disease.

Our results suggest a link between autoimmune thyroid disease and MS. There does not seem to be evidence for a temporal link between the two conditions as the age of onset of thyroid disease is the same in the general population as in the MS population. As well, the age of onset of MS is the same in subjects with and without thyroid disease. Therefore, this study does not provide evidence that the onset of one disease is the harbinger of another.

The biological plausibility of an autoimmune pathophysiological link between MS and HT or Graves' disease is unproven. Speculatively, MS is a disease characterized by activated T cells. Activated T cells produce a milieu of cytokines, notably IFN-γ. IFN-γ has been hypothesized to induce the autoimmune process observed in Hashimoto's disease. Therefore, the increased availability of activated T cells in MS may cause an increase of Hashimoto's disease in MS patients. The link between Graves' disease and MS is less clear, however experimental observations using Campath-1H may provide clues. Campath-1H is a molecule that targets the CD52 antigens found on lymphocytes and monocytes. It causes the immune response to change from the Th1 phenotype, thus suppressing MS disease activity [28] but permitting the generation of antibody-mediated thyroid autoimmunity. A significant increase in the rate of development of Graves' disease in MS patients was noted after treatment with Campath-1H [28], but Graves' disease has not been reported in over 600 patients treated with Campath-1H for various other autoimmune disorders including rheumatoid arthritis and psoriasis [28]. Further study along this direction may reveal the reason for the increased co-occurrence between Graves' disease and Multiple Sclerosis.

## Conclusion

Hashimoto's disease, Graves' disease and multiple sclerosis are autoimmune disorders that may share similar mechanisms for the pathogenesis of the dysregulation of their self immunity. This study has shown that there is a significant co-occurrence in patients with MS and Graves' disease, and a marginal co-occurrence in patients with MS and HT disease. There appears to be no predilection for earlier age of onset for either MS or the thyroid disorders and there appears to be no gender preference in the co-occurrence of the diseases. Reasons for their increased co-occurrence are discussed.
